# Resistance Training Combined with Balance or Gait Training for Patients with Parkinson's Disease: A Randomized Controlled Pilot Study

**DOI:** 10.1155/2022/9574516

**Published:** 2022-10-07

**Authors:** Johanna Theresia Biebl, Monica Azqueta-Gavaldon, Cornelia Wania, Olena Zettl, Matthias Woiczinski, Leandra Bauer, Claudia Storz, Kai Bötzel, Eduard Kraft

**Affiliations:** ^1^Department of Orthopaedics and Trauma Surgery, Musculoskeletal University Center Munich (MUM), University Hospital, LMU Munich, Marchioninistr. 15, Munich 81377, Germany; ^2^Department of Neurology, University Hospital, LMU Munich, Marchioninistr. 15, Munich 81377, Germany

## Abstract

**Background:**

Gait and balance disorders in patients with idiopathic Parkinson's disease (PD) lead to major mobility limitations. To counteract this, physical therapy such as gait, balance, or resistance training is applied. Integrative training methods, which combine these elements, could be particularly effective.

**Objective:**

The objective of this study is to evaluate and compare the effects of two integrative interventions on gait and balance of patients with PD.

**Methods:**

Twenty-six patients with PD received either resistance training in combination with gait training (gait resistance training, GRT) or resistance training in combination with balance training (stability resistance training, SRT) for six weeks. Gait and balance outcome parameters were assessed before, immediately after, and six weeks after the interventions. The primary outcome parameters were the functional reach test to evaluate balance and stride length to evaluate gait. Secondary outcomes included further gait analysis parameters, knee extension strength, the timed up and go test, and the six-minute walk test.

**Results:**

The functional reach test results were significantly better after the intervention in both groups. Stride length increased significantly only in the GRT group. Several further gait parameters and the six-minute walk test improved in the GRT group, and the increase in gait speed was significantly higher than in the SRT group. The SRT group performed better after the intervention regarding the timed up and go test and knee extension strength, the latter being significantly more improved than in the SRT group. At six-week follow-up, the improvement in functional reach was maintained in the SRT group.

**Conclusions:**

Integrative therapies, combining gait or balance training with resistance training, have specific positive effects in PD rehabilitation. More pronounced effects on gait parameters are achieved by GRT, while SRT has more impact on balance. Thus, the combination of both training methods might be particularly efficient in improving the mobility of PD patients.

## 1. Introduction

Parkinson's disease (PD) is a neurodegenerative disease with impairments increasing as the disease progresses [1, 2]. The cardinal symptoms are bradykinesia, rigidity, tremor, and postural instability. Consequently, gait and balance difficulties are common and lead to a reduced independence, decreased quality of life as well as increased fall risk [1, 3]. Physical exercise has been found to be very effective in improving physical and cognitive functional capacities in PD [4]. Resistance training in particular improves muscle strength, endurance, and physical function [5, 6]. Further important pillars of physical therapy in PD patients are balance and gait training, which have been proven to have positive effects on motor symptoms, balance, and gait [7]. Integrative exercise methods, combining resistance training with balance or gait training, could further enhance the impact on the mobility of PD patients. Balance resistance training, also described as instability resistance training, is a method relatively widely used by healthy persons [8], but promising results have also been reported in PD patients [9]. Simultaneous resistance and gait training on a treadmill is a novel approach whose effects have, to our knowledge, not yet been evaluated in PD patients.

Therefore, the aim of this study was to investigate whether these two integrative interventions, resistance in combination with gait training and resistance in combination with balance training, are able to positively influence functional disability of PD patients.

## 2. Materials and Methods

This pilot study was designed as a randomized, controlled, monocentric, equivalent trial with two parallel interventional groups. The participants were randomized into different intervention groups according to block-randomization with 1 : 1 allocation [10]. The randomization process was conducted by an independent person using an online randomization tool (https://www.randomization.com), applying five blocks of four subjects and one block of six subjects. Patients could not be blinded to group allocation.

### 2.1. Participants

Patients diagnosed with PD according to the Queen Square Brain Bank criteria [11] who were in a moderately severe stage (Hoehn and Yahr stage II-III [12]) were recruited through two outpatient clinics at our university hospital. The exclusion criteria were an implanted deep-brain stimulator, other neurological disorders, cardiovascular diseases, psychiatric comorbidities, and any other diseases which were likely to affect gait. An experienced physician assessed eligibility for inclusion at the outpatient clinic of the Department of Physical Medicine and Rehabilitation at the Musculoskeletal University Center Munich (MUM). Participants were advised not to start other gait and postural control rehabilitation interventions during the duration of the study. PD medication was unchanged during the study (testing and training). All participants were on levodopa medication. The training sessions and clinical assessments were scheduled one to two hours after the first intake.

The study was approved by the research ethics committee of the medical faculty of the LMU Munich (IRB-Number: 247–09) and was conducted in accordance with the Declaration of Helsinki. Participants gave written informed consent prior to their participation. The study was registered with the German Clinical Trials Register (DRKS-ID DRKS00025340).

### 2.2. Intervention Protocol

The intervention programs did not differ regarding the number of sessions, duration, and weekly frequency (twelve sessions lasting 30 minutes over six weeks). Given the experimental character of the intervention and for a better quantification of therapeutic effects, the sessions remained constant in length and difficult throughout the duration of the study. The exercises were designed by an interdisciplinary team composed of a physician specialized in the treatment of PD, a physiotherapist with several years of experience in neurorehabilitation, and biomedical engineers with expertise in the biomechanics of gait. The interventions were delivered face-to-face and individually to the patient by the physiotherapist and took place at the outpatient clinic of the Department of Physical Medicine and Rehabilitation at the Musculoskeletal University Center Munich (MUM).

#### 2.2.1. Resistance in Combination with Balance Training

**Figure 1:**
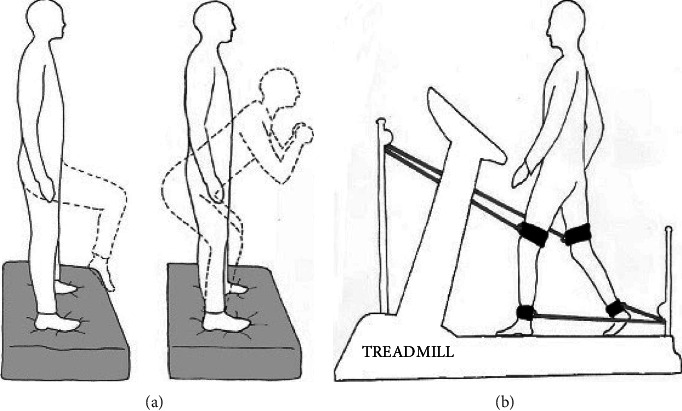
(a) Exercises performed by the stability resistance training (SRT) group. Participants carried out strength exercises on a balance-pad (grey). (b) Exercises carried out by the gait resistance training (GRT) group. Participants walked on a treadmill while elastic cables (gray) with cuffs (black) were attached to their legs to provide resistance during walking.

Stability resistance training (SRT), in the literature also referred to as instability resistance training (IRT), combines both resistance and balance exercises. This training is used in rehabilitation to strengthen the core or trunk muscles and improve coordination and motor control [8]. The patient performs resistance exercises while his/her postural stability is challenged [13]. In this study, a physiotherapist designed four different SRT exercises suitable for PD patients, in which the patients´ body mass was used as resistance, and a foam balance-pad (Airex®, Sins, Switzerland) or a balance-ball provided an unstable surface. Each exercise consisted of three sets of fifteen repetitions, with a resting period of two minutes between each exercise (Figure 1(a)).

The first exercise consisted of an adapted one-leg stand. Patients stood on the balance-pad with the feet hip distance apart and parallel to each other and slowly lifted one leg about 20 to 30 cm keeping the knee flexed, holding the leg in this position for 5 seconds before returning the raised foot to the floor.

During the second exercise, patients carried out adapted squads. Patients stood on the balance-pad with the feet hip distance apart and parallel to each other and slowly bent the knees and hip to lower the torso, then returning to the upright position.

The third exercise entailed adapted lunges: patients positioned one leg forward with the knee bent and foot flat on the ground while the other leg was positioned behind, and they slowly bent the knees to lower the torso.

During the fourth exercise, patients sat on a balance-ball with the arms stretched out upwards. They then slowly bent the hips to lean forward until touching the ground with the arms and finally returning to the upright position.

#### 2.2.2. Resistance in Combination with Gait Training

This exercise was designed to strengthen the trunk and leg muscles while training the gait. The patients walked on a treadmill while flexible cables attached to their lower extremities generated movement support or resistance (Robowalk Expander HP/Cosmos®, Nussdorf, Germany). For each leg, an elastic cable was mounted above the knee to the distal femoral region, providing tension in the anterior direction. This positioning of the cable served as an aid for hip flexion. In addition, another cable was attached above the ankles to the distal tibia region, providing resistance in the posterior direction (Figure 1(b)). Patients walked at their preferred velocity and, if possible, without holding onto the treadmill handrails.

### 2.3. Assessments

**Figure 2:**
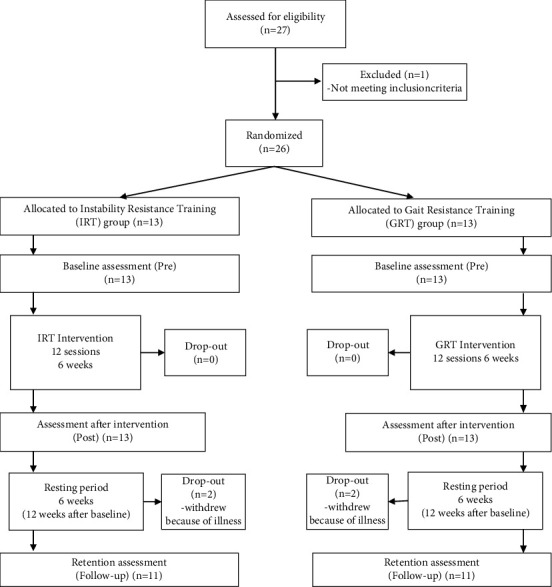
Study Design. Eligible participants were randomly allocated to one of the two groups and clinically assessed at the baseline. After (post) the six-week long intervention, participants were assessed again. Patients were called in again after a resting period of 6 weeks to undergo a follow-up assessment.

Each participant was evaluated for all primary and secondary outcome measures before the intervention (pretest), immediately after completion of the 6-week long exercises program (post-test), and after a six-week nonintervention period (follow-up), as shown in Figure 2.

The stride length was defined as the primary outcome of gait, and the functional reach test (FR) was set as the primary outcome of balance. Timed up and go (TUG), six-minute walking test (6-MWT), gait analysis parameters, motor symptoms of disease, and quality of life were secondary outcome measures.

#### 2.3.1. Functional Assessments

The functional abilities and balance of the participants using validated clinical tests were assessed. TUG and 6-MWT were used to evaluate mobility, balance, walking ability, and fall risk. To test stability and balance, the functional reach (FR) test was applied. Maximal isometric knee extension strength was also measured with a hand-held dynamometer.

#### 2.3.2. Gait Analysis

Spatio-temporal parameters of the gait cycle, as well as ground reaction forces were obtained through gait analysis performed on a treadmill with an embedded pressure plate (Rehawalk® Zebris, Isny, Germany). The most relevant parameters included in the analyses were gait velocity, normalized stride length, stride width and their variability, normalized cadence, trajectory of center of pressure, stance phase and double support phase times, and walking ratio [14]. Normalization of stride length and cadence [15] accounts for the dependence of these parameters on the patients' height. The walking ratio is a parameter that measures gait performance independently from the height of the subject and the walking velocity. The gait parameters were recorded at the patients' preferred speed, which they chose while the speed display was covered.

#### 2.3.3. Motor Symptoms

The severity of motor signs of PD was evaluated using Part III of the Unified Parkinson´s Disease Rating Scale (UPDRS–III). This validated scale is widely used and assesses signs such as rigidity, tremor, postural and gait stability, as well as movement variability and bradykinesia.

#### 2.3.4. Quality of Life

The Parkinson's Disease Questionnaire (PDQ-39) was used to assess the quality of life. This 39-item questionnaire consists of seven discrete scales: mobility, activities of daily living, emotional well-being, stigma, social support, cognition, and communication.

#### 2.3.5. Statistical Analysis

To test for normality and homoscedasticity of all outcome parameters recorded at different assessment times as well as their deltas (post and pre and follow-up and post), Shapiro–Wilk test and Levene's test were applied, respectively.

Parametric and nonparametric tests (*T*-tests and Wilcoxon signed-rank tests) were used to examine the within-group effects of the two interventions on all primary and secondary outcomes. One-way analysis of variance (ANOVA) was performed to compare the effects between the groups. Furthermore, to assess the clinical relevance of our findings, we calculated the effect size (ES), also known as Cohen's [8]. The ES values were classified as minor (0.20 ≤ ES < 0.50), moderate (0.50 ≤ ES < 0.80), and large (ES ≥ 0.80).

All results are expressed as the mean and standard deviation (M ± SD) for descriptive statistics.

## 3. Results

Twenty-six participants were randomly assigned to two groups. All of the patients successfully completed the interventions without need to modify such interventions. The average assistance rate was 92%. Four participants dropped out due to mild sickness before being assessed at follow-up (see Figure 2). No adverse events related to the interventions occurred. In none of the patients, off-phases or levodopa-induced dyskinesias were observed during testing and training. There were no differences between the groups concerning age, disease severity and duration, female-to-male ratio, as well as symptoms at the baseline. The demographic characteristics and baseline values of the study participants are presented in Table 1. An overview of the effects of the therapies can be found in Table 2.

### 3.1. Primary Outcomes

Both groups were able to reach further in the FR test (*p* < 0.05). These changes were not significantly different between the two groups (*F* (1, 25) = 0.12, *p*=0.74).

In the GRT group, the normalized stride length was longer after the intervention (*p* < 0.01), while in the SRT group, no significant changes regarding stride length were found.

### 3.2. Secondary Outcomes

Participants in the SRT group needed less time to perform the TUG test (*p* < 0.05) after the intervention. Moreover, maximal strength in the knee extension was also significantly greater after intervention for the SRT group (*p* < 0.05), and this change was superior compared to the GRT group (*F* (1, 25) = 31.262, *p* < 0.05). Following the interventions, the GRT group showed an improvement in 6-MWT (*p* < 0.05). *F* (1, 25) = 0.74, *p*=0.40). Furthermore, patients from the GRT group improved regarding their gait parameters. These patients achieved an increase in walking speed (*p* < 0.01), which was significantly higher than in the SRT group (*F* (1, 25) = 7.701, *p* < 0.05). A correlation analysis demonstrated that the increase in stride length did not correlate with an increase in velocity but instead with a decrease in cadence (*rho* (11) = −71, *p* < 0.001). The walk ratio improved moderately (*p*=0.21), and stride length variability was significantly reduced (*p* < 0.05). Moreover, the GRT group showed an increase in the normalized foot roll line (*p* < 0.05). In terms of gait parameters, the SRT group did only experience an improvement in the variability of the step width (*p* < 0.05).

### 3.3. Motor Symptoms

Regarding the motor symptoms measured with the UPDRS-III, both groups showed slight improvement after the intervention (SRT: ΔM = −2.46; GRT: ΔM = −2.38). However, within-group and between-group comparisons did not yield statistically significant results.

### 3.4. Quality of Life

Only the SRT group demonstrated a trend towards improvement of well-being as evaluated by the PDQ-39 questionnaire (*p*=0.08): questions concerning activities of daily living were answered more positively (*p* < 0.01), and a significant improvement in the cognition scale (*p* < 0.05) was found. The GRT group did not show any significant changes after the training.

### 3.5. Follow-Up

At 6-week follow-up, the FR test remained improved in the SRT group (*p* < 0.5). The step width that showed no statistical difference in the postintervention measurement decreased significantly at the follow-up measurement (*p* < 0.1) in the GRT group. Regarding all other parameters, no statistically significant changes were found.

## 4. Discussion

The aim of the study was to investigate whether two integrative resistance interventions have positive effects on functional disability. The results of our randomized pilot study indicate that both groups got benefited from the respective interventions, with improvements in different functional domains.

Patients carrying out SRT showed an improvement regarding balance and mobility as indicated by the FR and TUG assessments. Our results support previous research, showing SRT to improve balance and functional performance [13] including in the elderly [16] and in PD patients [9]. Moreover, these patients gained muscular strength related to knee extension. This result might seem predictable since it has been widely shown that resistance training has a positive effect on knee extension strength in PD patients [17]. However, unlike most of the resistance exercises which are machine-based and make use of external weights, our patients used their own body mass as resistance. The exercises employed in the present study are beneficial for improving posture and lower limb strength and are simple enough to be carried out at home.

Existing data on the influence of conventional resistance training on gait are inconsistent. While one meta-analysis showed an improvement in 6MWT through resistance training [7], another meta-analysis found no such effect [6]. In our study, it was not possible to assess with certainty whether the combination of training forms in the sense of SRT has a relevant impact on gait. Although there was a positive influence on the step width variance, other parameters such as the 6MWT were not influenced in our sample.

The impact of the GRT intervention on gait was more conclusive. The patients of this treatment group showed a faster and more stable and physiological gait, characterized by longer strides, a reduced stride length variability, a smaller step width (on follow-up), a longer center of pressure trajectory, or foot roll line. A longer foot roll line renders into a greater dorsiflexion of the ankle. An improvement in balance measured by functional reach was also observed. Gait training on a treadmill is currently a part of the standard therapeutic routine for gait rehabilitation in PD, and several studies have shown the benefits [18] of such interventions. Nevertheless, in contrast to a previous study [19], our patients showed an improvement of the range of motion ankle with a higher dorsiflexion, which suggests a beneficial effect in PD cardinal symptoms such as rigidity and bradykinesia. Moreover, a proper dorsiflexion of the ankle reduces the possibility of stumbling and thus the risk of falling. The resistive component of the exercise might have played a role in these findings. Although the resistance used did not suffice to improve muscle strength, it might have had a positive effect on motor control of the dorsiflexor muscles. Moreover, the exerted resistive force was not constant during the gait cycle. Instead, its magnitude and direction changed during the different stages of the gait cycle. In this manner, the exerted resistive forces may have acted as proprioceptive cues. Previous studies have shown that cues, either auditory (rhythmic beeps or metronome) or visual (rhythmically flashing lights or marks on the floor), are effective at normalizing Parkinsonian gait. In addition, they are helpful against the freezing of the gait phenomenon, typically seen in PD patients [20]. On these grounds, visual cues have been incorporated in gait training with encouraging results [21]. Proprioceptive cues have also shown to have a positive effect in cadence and stride length in PD patients [22]. In addition, perturbation training with three-dimensional tilting movements of the treadmill surface was able to influence motor symptoms and gait and postural stability in PD patients more positively than conventional treadmill training [23].

Most positive effects were not sustained in both training groups at six-week follow-up, indicating that a continuous training session would be necessary to maintain lasting effects, taking into account the nature of the disease.

Our study has several limitations. The posthoc statistical power achieved with the number of patients in each group (*n* = 13) was greater than 90%. However, the number of patients is still too small to be representative of the PD patient population, especially since recruited patients had only mild to moderate symptoms. Studies with larger patient groups including PD patients with more severe symptoms are necessary to validate our results. Furthermore, the design of the study does not allow conclusions on whether GRT is superior to conventional treadmill training as there was no such control group. Sustainability of treatment effects was investigated with a follow-up assessment which was carried out 6 weeks after the end of the intervention. To analyze long-term effects, an additional follow-up evaluation after a longer period of time would have been necessary. Moreover, it is important to consider the intrinsic limitations of assessing gait on a treadmill, as walking on a treadmill usually results in lower velocity and shorter steps compared to walking on the ground.

## 5. Conclusions

Integrative therapies composed of resistance training in combination with either balance or gait training have positive effects in PD rehabilitation. More pronounced effects on gait parameters were achieved by GRT in our study, while SRT had more impact on balance. Thus, we believe a combination of these two integrated trainings would have synergetic mechanisms and address different functional impairments of PD patients efficiently.

## Figures and Tables

**Table 1:** Demographic and clinical information of the participants.

	SRT mean (±SD) (*n* = 13)	GRT mean (±SD) (*n* = 13)	Group differences *p* value
Demographic information			
Age (years)	71.9 (7.6)	71.8 (8.5)	0.981
Ratio women/men	6/7	7/6	0.500

Clinical parameters			
Duration of disease (years)	6.00 (4.63)	6.54 (3.64)	0.745
Stage of disease (H&Y)	2.35 (0.47)	2.39 (0.42)	0.828
UPDRS-III (score 0–56)	45.31 (12.32)	44.38 (11.52)	0.845
Tibia length (cm)	41.42 (2.91)	41.19 (2.63)	0.842

**Table 2:** Comparison of the effects of stability resistance training and gait resistance training.

	SRT						GRT							
	Pre (*n* = 13)		Post (*n* = 13)		Follow-up (*n* = 11)		Pre (*n* = 13)		Post (*n* = 13)		Follow-up (*n* = 11)		Effects between the groups^§^	
	Mean	(±SD)	Mean Δ^†^	ES	Mean Δ^‡^	ES	Mean	(±SD)	Mean Δ^†^	ES	Mean Δ^‡^	ES	*F*	*p* value
UPDRS-III (0–56)	45.31	(12.32)	−2.46	−0.24	−1.73	−0.22	44.38	(11.52)	−2.38	−0.26	−2.36	−0.36	0.001	0.984
FR (cm)	25.31	(6.613)	+3.62^*∗∗*^	+0.52	+2.91^*∗∗*^	+0.52	25.54	(4.79)	+2.77^*∗∗*^	+0.50	−1.27	−0.36	0.118	0.735
TUG (s)	8.92	(1.61)	−1.00^*∗∗*^	−0.55	−0.37	−0.36	9.58	(2.52)	−0.46	−0.21	+0.21	+0.20	0.453	0.507
6 MWT (m)	497.38	(45.78)	+16.12	+0.24	+19.14^*∗*^	+0.52	429.38	(87.35)	+37.46^*∗∗*^	+0.62	+5.18	+0.10	0.744	0.397
Perceived exertion (0–10)	5.25	(1.71)	−0.58	−0.30	+0.73	+0.46	5.08	(2.14)	−0.08	−0.05	+0.64	+0.30	0.646	0.429
Max. force (kp)	24.50	(8.89)	+2.17^*∗∗∗*^	+0.80	+1.36	+0.33	19.71	(13.00)	0.00	0.00	−0.18	−0.04	31.262	0.030^*∗∗*^
Stride length (cm)^n^	75.99	(17.94)	+2.23	+0.33	+2.84	+0.14	64.51	(22.64)	+12.00^*∗∗∗*^	+1.09	−3.09	−0.28	3.066	0.093
Stride length variability (cm)	5.43	(3.99)	−1.87^*∗*^	−0.49	+0.56	+0.25	3.33	(1.22)	-0.82^*∗∗*^	-0.61	0.00	+0.01	0.897	0.353
Step width (cm)	12.18	(4.95)	−0.32	−0.14	−0.55	−0.30	13.91	(5.03)	-0.40	-0.18	−1.17^*∗∗*^	−0.63	0.007	0.933
Step width variability (cm)	2.43	(0.67)	−0.44^*∗∗*^	−0.54	+0.38	+0.74	1.41	(0.71)	+0.13	+0.19	−0.28	−0.22	3.671	0.067
Stance phase (%)	67.18	(2.68)	+0.21	+0.06	−0.54	−0.19	69.24	(2.75)	-0.52	-0.27	+0.26	+0.14	0.417	0.525
Double support phase (%)	35.89	(5.68)	+0.25	+0.05	−1.88	−0.35	35.88	(11.92)	+1.61	+0.13	0.00	0.00	0.151	0.701
Cadence (steps/min)^n^	94.41	(15.08)	−2.41	−0.17	+4.58	+0.35	98.35	(18.00)	-3.01	-0.18	+2.04	+0.31	0.010	0.922
Walking speed (km/h)	2.11	(0.53)	0.00	0.00	+0.23^*∗*^	+0.48	1.82	(0.45)	+0.36^*∗∗∗*^	+1.33	−0.04	−0.08	7.701	0.011^*∗∗*^
Walk ratio (mm/(step/min))^n^	4.21	(1.44)	+0.27	+0.23	−0.18	−0.23	3.55	(1.76)	+0.59^*∗*^	+0.43	−0.28	−0.54	0.381	0.543
Foot roll line (cm)^n^	20.51	(3.55)	+0.71	+0.17	+0.47	+0.23	19.34	(4.69)	+1.7.9^*∗∗*^	+0.74	−0.16	−0.12	0.646	0.429

^*∗*^ *p* < 0.1 (trend); ^*∗∗*^*p* < 0.05; ^*∗∗∗*^*p* < 0.01; Δ^†^delta post-pre; Δ^‡^delta follow-up - post;^ §^post-pre; ES: effect size; ^n^normalized. SRT: stability resistance training; GRT: gait and resistance training; UPDRS: unified Parkinson's disease rating scale; FR: functional reach test; TUG: timed up and go test; 6 MWT: 6-minute walk test.

## Data Availability

The data used to support the findings of this study are included within the article.
